# Induction of Type I Interferons by Therapeutic Nanoparticle-Based Vaccination Is Indispensable to Reinforce Cytotoxic CD8^+^ T Cell Responses During Chronic Retroviral Infection

**DOI:** 10.3389/fimmu.2018.00614

**Published:** 2018-04-23

**Authors:** Torben Knuschke, Olga Rotan, Wibke Bayer, Sebastian Kollenda, Julia Dickow, Kathrin Sutter, Wiebke Hansen, Ulf Dittmer, Karl S. Lang, Matthias Epple, Jan Buer, Astrid M. Westendorf

**Affiliations:** ^1^Institute of Medical Microbiology, University Hospital Essen, University of Duisburg-Essen, Essen, Germany; ^2^Institute of Inorganic Chemistry, Center for Nanointegration Duisburg-Essen (CeNIDE), University of Duisburg-Essen, Essen, Germany; ^3^Institute of Virology, University Hospital Essen, University of Duisburg-Essen, Essen, Germany; ^4^Institute for Immunology, University Hospital Essen, University of Duisburg-Essen, Essen, Germany

**Keywords:** nanoparticles, therapeutic vaccine, chronic retrovirus infection, T cell immunity, type I interferons, interferon alpha

## Abstract

T cell dysfunction and immunosuppression are characteristic for chronic viral infections and contribute to viral persistence. Overcoming these burdens is the goal of new therapeutic strategies to cure chronic infectious diseases. We recently described that therapeutic vaccination of chronic retrovirus infected mice with a calcium phosphate (CaP) nanoparticle (NP)-based vaccine carrier, functionalized with CpG and viral peptides is able to efficiently reactivate the CD8^+^ T cell response and improve the eradication of virus infected cells. However, the mechanisms underlying this effect were largely unclear. While type I interferons (IFNs I) are considered to drive T cell exhaustion by persistent immune activation during chronic viral infection, we here describe an indispensable role of IFN I induced by therapeutic vaccination to efficiently reinforce cytotoxic CD8^+^ T cells (CTL) and improve control of chronic retroviral infection. The induction of IFN I is CpG dependent and leads to significant IFN signaling indicated by upregulation of IFN stimulated genes. By vaccinating chronically retrovirus-infected mice lacking the IFN I receptor (IFNAR^−/−^) or by blocking IFN I signaling *in vivo* during therapeutic vaccination, we demonstrate that IFN I signaling is necessary to drive full reactivation of CTLs. Surprisingly, we also identified an impaired suppressive capability of regulatory T cells in the presence of IFNα, which implicates an important role for vaccine-induced IFNα in the regulation of the T cell response during chronic retroviral infection. Our data suggest that inducing IFN I signaling in conjunction with the presentation of viral antigens can reactivate immune functions and reduce viral loads in chronic infections. Therefore, we propose CaP NPs as potential therapeutic tool to treat chronic infections.

## Introduction

Clearance of chronic viral infections, such as hepatitis B virus and human immunodeficiency virus (HIV) infection, is one of the major goals of immunotherapeutic vaccination. Cytotoxic CD8^+^ T cells (CTL) are crucial for eliminating virus infected cells and controlling acute infection. One mechanism that leads to viral persistence is virus-induced dysfunctionality of these CD8^+^ T cells mediated by regulatory T cells (Treg) or the induction of inhibitory signaling pathways such as PD-1/PD-L1 (1, 2). To overcome these hurdles and effectively reactivate the dysfunctional CTL response immune-based therapies have been considered as efficient treatment of chronic viral infections (3–5). Hence, the development of new therapeutic approaches to reactivate immune responses in an immunosuppressed environment is the focus of recent science (5, 6). In this regard, we have developed calcium phosphate (CaP) nanoparticles (NPs) as a novel tool for immunotherapeutic purposes. CaP NPs can be functionalized with several kinds of small molecules and act as carriers across cell membranes (7). In recent studies, we showed that the functionalization of CaP NPs with the toll-like receptor (TLR) 9 ligand CpG oligonucleotide (ODN) and viral antigens facilitates a strong activation of dendritic cells (DCs) and virus-specific T cell responses after immunization (8, 9). Experiments in the murine Friend retrovirus (FV) model revealed that therapeutic vaccination of chronically infected mice leads to a reactivation of CTL responses and a significant drop in viral loads (10, 11). So far, it is not well defined which pathway is predominantly responsible for the reactivation of CTLs in our experimental system. CpG ODNs induce several pro-inflammatory cytokines such as interleukin-12 (IL-12), tumor necrosis factor-α (TNF-α), as well as IFNs I *via* TLR 9 signaling (12). IFNs I represent a powerful cytokine family that includes more than 10 subtypes of IFNα, and IFNβ, all binding to the same IFNα receptor (13). They have clear direct effects by inducing anti-viral enzymes, but are also known to affect functional properties of immune cells (14). For example, IFNs I are important for the activation of virus-specific T cell responses by enhancing T cell priming (15) and for increasing their functionality including the cytotoxic capacity of CTLs (16, 17). Interestingly, during retroviral HIV or FV infection, the endogenous IFN I response is rather weak (18, 19). Therefore, IFNα-based therapies have been established for anti-viral treatment of HIV infection (20–22).

Nevertheless, the protectiveness of IFN I is also seen critically because of their inhibitory potential on immune responses. Administration of IFNα2a was shown to prevent systemic simian immunodeficiency virus infection (23). On the other hand, constant expression of IFN I during chronic viral infection or continued IFNα treatment can lead to desensitization linked to disease progression (23–25). Therefore, it seems that the quantity as well as the timing of IFN I delivery may be important for the success of immunotherapy.

In this study, we determined the therapeutic effect of CpG functionalized CaP NP induced IFN I on the anti-viral T cell response during chronic Friend retroviral infection. FV is an oncogenic retroviral complex that induces lethal erythroleukemia in susceptible mouse strains. However, resistant strains show a robust immune response that prevents leukemia, but develop a chronic infection due to virus-induced immune suppression and T cell dysfunction (26). It was recently reported that poly(I:C) treatment of mice during acute FV infection improves the functionality of virus-specific T cells *via* the release of IFNα (27). However, it is not clear so far whether the exogenous induction of IFN I can contribute to the reactivation of the dysfunctional T cell response during chronic FV infection. In the current study, we show that the success of therapeutic vaccination of chronic FV infected mice was dependent on the induction of IFN I. As important underlying mechanisms, we identified a significant effect of IFN I on the inhibitory capacity of CD4^+^ Treg and the cytotoxic capacity of CTLs.

## Materials and Methods

### Mice

C57BL/6 mice were purchased from Envigo Laboratories (Envigo CRS GmbH, Rossdorf, Germany). IFNAR deficient mice (IFNAR^−/−^) on C57BL/6 background were described previously (28). DEREG (DEpletion of REGulatory T cells) mice [expressing eGFP and diphtheria toxin receptor under the control of the forkhead box P3 (Foxp3) promoter] on C57BL/6 background were described by Lahl et al. (29). All mice used in the experiments were 8–10 weeks old at time point of infection and housed under specific pathogen-free conditions in the Laboratory Animal Facility of the University Hospital Essen.

### Cells and Cell Culture

A murine fibroblast cell line from *Mus dunni* (30) was maintained in Roswell Park Memorial Institute (RPMI) medium containing 10% endotoxin free fetal calf serum (FCS) and 50 µg mL^−1^ penicillin/streptomycin. Cell lines were maintained in a humidified 5% CO_2_ atmosphere at 37°C.

### TLR-Ligand and Viral Peptides

The phosphorothioate-modified class B CpG 1826 was purchased from Eurofins MWG Operon. The FV protein derived Gag and gp70 peptide sequences containing MHC I and MHC II epitopes were synthesized with the following sequences: GagL_85–93_, CCLCLTVFL; gp70_123–141_, EPLTSLTPRCNTAWNRLKL (JPT Peptide Technologies GmbH). The cysteines in the GagL peptide sequence was exchanged by aminobutyric acid.

### Preparation of Functionalized NPs

The NPs were prepared by fast mixing of an aqueous solution of calcium nitrate (6.25 mM) with an aqueous solution of diammonium hydrogen phosphate (3.74 mM). The pH value of both solutions was adjusted beforehand to 9 with NaOH (0.1 M). Mixing was achieved by rapidly pumping both solutions in a 1:1 ratio into a glass vessel. Immediately after mixing, 1 mL of the CaP dispersion was mixed with 0.2 mL of an aqueous solution of CpG (63 µM = 400 µg mL^−1^) in an Eppendorf tube.

For preparation of triple-shell NPs, 50 µL of the GagL or gp70 peptide (1 mg mL^−1^), followed by 0.5 mL of calcium nitrate solution (6.25 mM) and then 0.5 mL of diammonium hydrogen phosphate solution (3.74 mM) were added to the dispersion of single-shell NPs (CaP/CpG/GagL/CaP/CpG; CaP/CpG/gp70/CaP/CpG) (8, 31).

In the next step, the triple-shell NPs were removed from the supernatant by ultracentrifugation for 30 min at 66,000 g. Afterward, the centrifuged NPs were redispersed in water (in one quarter of the initial volume) with an ultrasonic processor (UP50H, Hielscher, Ultrasound Technology; cycle 0.8, amplitude 60%) for 10 s. The final concentrations of CpG and GagL/gp70 peptide were 20.6 µM and 40.8 µg mL^−1^, respectively (10).

All inorganic salts were of pro analysi quality. Ultrapure water (Purelab ultra instrument from ELGA) was used for all preparations. All formulations were prepared and analyzed at room temperature. The particles were characterized by scanning electron microscopy (ESEM Quanta 400) with palladium-sputtered samples. Dynamic light scattering was performed with a Zetasizer nanoseries instrument (Malvern Nano-ZS, λ = 532 nm). The particle size data refer to scattering intensity distributions (*z*-average). Calcium concentrations were determined by atomic absorption spectroscopy (AAS; M-Series AA spectrometer; ThermoElectron Corporation, Schwerte, Germany) after dissolution of the particles in hydrochloric acid.

Scanning electron microscopy showed spherical NPs with a typical diameter of 80 nm. Dynamic light scattering gave a hydrodynamic radius of 200–300 nm (pointing to some degree of agglomeration) and a zeta potential of −21 mV. The calcium concentration in the dispersions was 67 µg mL^−1^ Ca^2+^ (by AAS), resulting in a CaP concentration of 167 µg mL^−1^ [assumption of the stoichiometry of hydroxyapatite; Ca_5_(PO_4_)_3_OH]. Together with the particle diameter from SEM, this results in a particle concentration of 2.0 × 10^11^ particles mL^−1^.

### Antibodies and Flow Cytometry

The monoclonal antibodies αCD4 (clone RM4-5), αCD8 (clone 53-6.7), αCD40 (clone 3/23), αCD80 (clone 16-10A1), αCD86 (clone GL1), and αCD43 (clone 1B11) were obtained from BD Biosciences Pharmingen. αGzmB antibody (clone GB12, Invitrogen) was used for intracellular granzyme B (GzmB) staining. The αFoxp3 (clone FJK-16s) antibody was purchased from eBioscience. Intracellular staining with αFoxp3 and αGzmB was performed as described previously (10). αIFNy (clone XMG1.2), αIL-2 (clone JES6-5H4), and αTNFα (clone MP6-XT22) antibodies were obtained from Biolegend. To detect Friend virus-specific CD8^+^ T cells, a PE-conjugated recombinant MHC-class I H2-D^b^ tetramer (Beckman Coulter) specific for FV GagL peptide was used (32). Data were acquired by using an LSR II instrument using DIVA software (BD Biosciences Pharmingen).

### Isolation of DCs

For the isolation of DCs, splenic (SP) tissue was first cut into small pieces and then treated with 1 mg mL^−1^ collagenase type D (Roche Diagnostics GmbH) and 10 mg mL^−1^ deoxyribonuclease I type II (SigmaeAldrich Chemie GmbH, St. Louis, MO, USA) diluted in phosphate-buffered saline (PBS) with 2% FCS and 2 mM ethylenediaminetetraacetic acid (EDTA), incubated for 45 min at 37°C, and mechanically minced and filtered through a 100 mm cell strainer. Cells were washed with PBS containing 2% FCS and 2 mM EDTA. CD11c^+^ cells were positively selected on MACS columns according to the manufacturer’s instructions (Miltenyi Biotec).

### IFNα Detection

Dendritic cells were isolated and 8 × 10^5^ cells per well were stimulated with 40 µL of CaP NPs containing CpG and viral peptides or polystyrene sulfonate (PSS) and viral peptides. Supernatant was collected 4 h later and IFNα levels were quantified using the polystyrene bead-based Luminex Assay (R&D Systems) according to the manufacturer’s instructions. The assay was run on a Luminex 200 instrument (Luminex Corporation).

### RNA Isolation and Quantitative Real-Time PCR

RNA was obtained from popliteal lymph nodes (LNs) of vaccinated mice using the RNeasy Kit (Qiagen). cDNA was synthesized with M-MLV Reverse Transcriptase (Promega) and OligodT mixed with Random Hexamer primers (Invitrogen). Real-time RT-PCR was performed using the SYBR Green PCR kit (Fermentas/Thermo Fisher Scientific) and specific primers for IFNα (5′ ATGGCTAGGCTCTGTGCTTTCC; 3′ AGGGCTCTCCAGACTTCTGCTCTG) and RPS9 (5′ CGCAGGCGCAGACGGTGGAAGC; 3′ CGAAGGGTCTCCGCGGGGTCACAT) on an ABI PRISM cycler (Applied Biosystems, Life Technologies). Relative RNA levels were determined with included standard curves for each individual gene and further normalization to the housekeeping gene RPS9. Primers for MX1, IRF7, and USP18 were purchased from Qiagen.

### *In Vitro* Stimulation of CD8^+^ T Cells

Murine bone-marrow derived DCs (BM-DCs) were generated as described before (33) and were incubated with 0.1 µg mL^−1^ FV GagL peptide (34) for 90 min at 37°C. SP TCRtg CD8^+^ T cells were isolated by MACS technology (Miltenyi Biotec). For antigen-specific activation of CD8^+^ T cells, 2.5 × 10^5^ TCRtg CD8^+^ T cells (32) were co-cultured with 0.5 × 10^5^ peptide-loaded BM-DCs for 72 h and additionally stimulated with different concentrations of universal IFNα (PBL Assay Science). Afterward, intracellular cytokine staining of GzmB, IFNγ, IL-2, and TNFα was performed.

### Friend Virus and Chronic Infection

A lactate dehydrogenase-elevating virus (LDV) B-cell-tropic, polycythemia-inducing Friend virus-complex was obtained from BALB/c mouse spleen cell homogenate 14 days postinfection (35) to establish chronic infection. To induce chronic FV infection, naïve FV resistant CB57BL/6 mice were challenged with 15,000 spleen focus forming units (SFFU). For acute FV infection mice were infected with 20,000 SFFU of an LDV free stock.

### Vaccination of Mice

C57BL/6 mice or IFNAR^−/−^ mice were vaccinated subcutaneously either with 100 µL PBS or CaP NPs functionalized with CpG/GagL/gp70 (10.3 µM CpG and 40.8 µg mL^−1^ GagL/gp70 peptide; CaP/CpG/GagL/gp70/CaP/CpG) in both hind footpads (50 µL each). 5 h postvaccination, mice were sacrificed for RNA isolation from skin and LN samples.

To determine the maturation of DCs after therapeutic NP vaccination, mice were immunized subcutaneously with 100 µL PBS or functionalized CaP NPs. 6 h later, popliteal LNs were harvested. Cells of LNs were isolated and used for further analysis.

For the treatment of chronic FV infection, mice were immunized subcutaneously with 100 µL PBS or functionalized CaP NPs 6 weeks post-FV infection as described before (10).

### IFNAR Blockade

To blockade IFN I signaling, mice were injected intraperitoneally with 250 µg anti-mouse IFNAR-1 blocking antibody (Clone MAR1-5A3, Hölzel Diagnostika) diluted in PBS 1 day before and 2 days after vaccination with functionalized CaP NPs.

### IFN-γ ELISpot Assay

Interferon-γ-producing cells were evaluated by ELISpot with a mouse ELISPOTPLUS kit (Mabtech AB). Cells were collected 7 days after vaccination from the spleen of FV infected DEREG mice by rinsing the spleen with an erythrocyte lysis buffer and washing with PBS containing 2% of FCS and 2 mM EDTA. Restimulation of 2.5 × 10^5^ cells was done with 5 µg mL^−1^ GagL_85–93_ or gp70_123–141_ peptide in a 96-well ELISpot plate for 24 h. IFN-γ-producing cells were stained according to the manufacturer’s instructions. After the plate was dried, the number of spots was counted by an AID ELISPOT reader using ELISPOT 6.0 software (Mabtech).

### Infectious Center (IC) Assay

Determination of viral loads by IC assay was performed as described previously (36). Spleens were cropped and rinsed with RPMI containing 10% FCS and 50 µg mL^−1^ penicillin/streptomycin. Spleen cells were counted and serially diluted before seeding them onto *Mus dunni* tail fibroblast cells and incubated under standard tissue culture conditions for 3 days, fixed with ethanol and labeled with the primary F-MuLV Env-specific MAb 720 (37). After washing, a secondary horseradish peroxidase (HRP)-conjugated rabbit anti-mouse Ig antibody (Dako) was added. Foci representing ICs were detectable after adding of aminoethylcarbazole (Sigma-Aldrich) as substrate for HRP. Foci were counted and ICs/spleen values were calculated.

### Suppression Assay

CD4^+^ Foxp3^+^ (eGFP^+^) Tregs were separated from spleens of non-infected or chronically FV infected DEREG mice using a FACSAria II cell sorter (BD Biosciences). CD4^+^ CD25^hi^ cells were sorted to obtain Treg cells from IFNAR^−/−^ mice. CD8^+^ T cells were purified from spleens of naive C57BL/6 or IFNAR^−/−^ mice with the CD8^+^ T Cell Isolation Kit (Miltenyi Biotec) and labeled with eFluor670 according to the manufacturers protocol (eBioscience); these cells served as responder T cells. CD8^+^ responder T cells (1 × 10^5^) were either cultured alone or co-cultured with CD4^+^ Foxp3^+^ (eGFP^+^) Tregs (5 × 10^4^) or CD4^+^ CD25^hi^ cells, respectively, for 3 days in the presence of 1 µg mL^−1^ anti-CD3 (2C11; BD Biosciences) and were additionally stimulated with different concentrations of universal IFNα (PBL Assay Science). Irradiated splenocytes from naïve C57BL/6 or IFNAR^−/−^ mice were added and served as antigen-presenting cells (3 × 10^5^). Inhibition was calculated by normalizing the proliferation of CD8^+^ T cells co-cultured with Tregs to CD8^+^ T cell proliferation without Tregs.

### Statistical Analysis

Statistical analysis was performed by using Student’s *t*-test or one-way ANOVA to compare multiple groups using Bonferroni’s multiple comparison test. Data analysis was performed using Prism 6.0 software (GraphPad). Statistical significance was set at the level of *p* < 0.05.

### Ethics Statement

This study was carried out in accordance with the recommendations of the Society for Laboratory Animal Science (GV-SOLAS) and the European Health Law of the Federation of Laboratory Animal Science Associations (FELASA). The protocol was approved by the North Rhine-Westphalia State Agency for Nature, Environment and Consumer Protection (LANUF), Germany (Permit Number: Az.: 84-02.04.2014.A290).

## Results

### Induction of IFNs I by CpG Functionalized NPs

**Figure 1:**
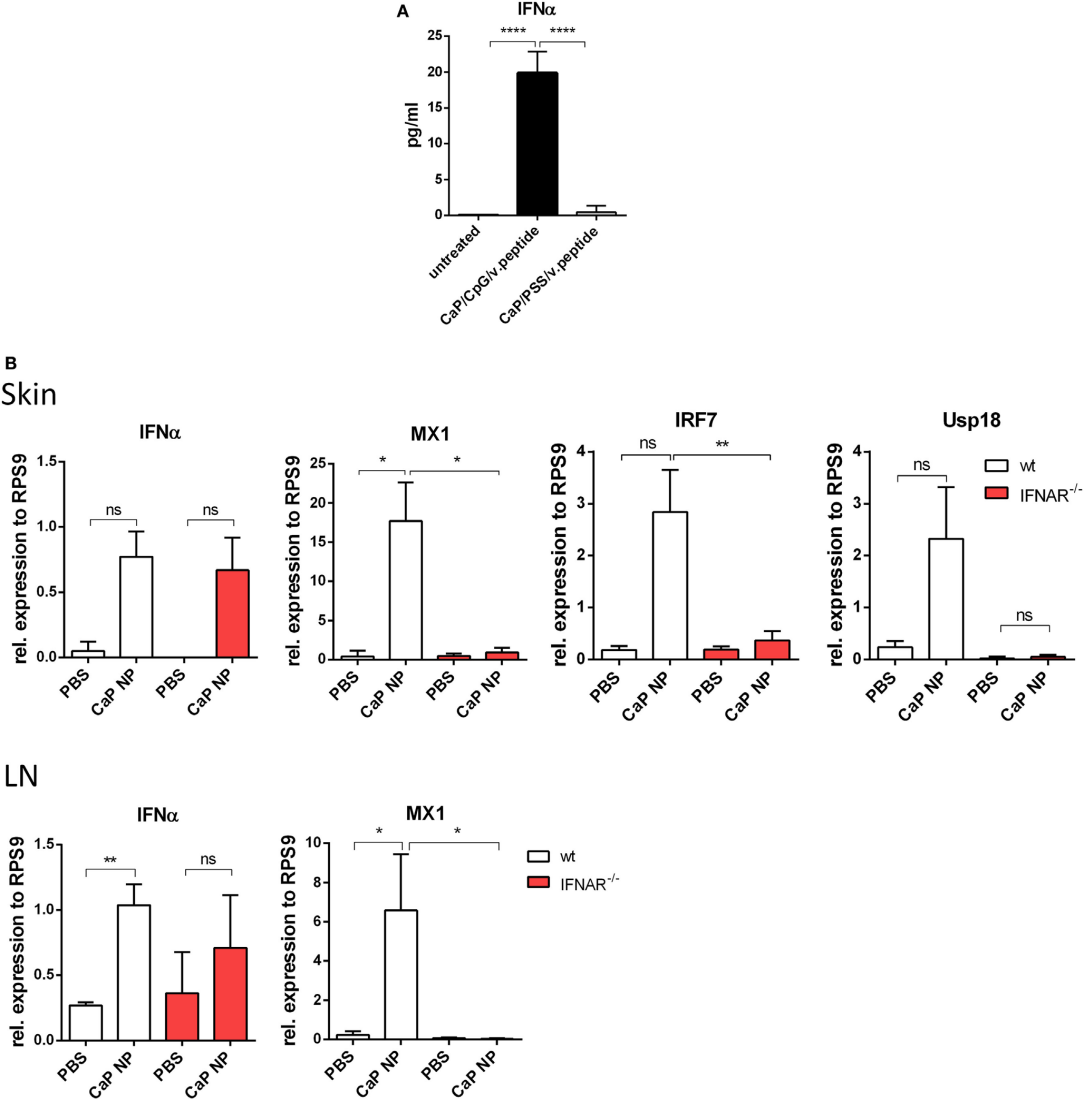
Induction of type I interferon (IFN I) signaling by CpG functionalized calcium phosphate (CaP) nanoparticles (NPs). **(A)** CD11c^+^ cells were isolated from the spleen and incubated with CpG functionalized CaP NPs or CaP NPs functionalized with non-immunogenic polystyrene sulfonate. Concentrations of IFNα in cell culture supernatants were analyzed 4 h later by Luminex technology, *n* = 6. Statistical analysis was performed by ANOVA with Bonferroni’s multiple comparisons test (****p < 0.0001). **(B)** Expression of IFNα and the interferon regulated genes (IRF) MX1, IRF7, and Usp18 were analyzed by quantitative real-time PCR in the skin and the popliteal lymph node 5 h postvaccination, *n* = 6. The results of two independent experiments are shown. Bars represent mean ± SEM. Statistical analysis was performed by Student’s *t*-test (*p < 0.05; **p < 0.01).

Dendritic cells are known to release significant amounts of IFN I when stimulated with TLR ligands such as poly(I:C) or CpG ODNs. In previous studies, we described triple-shell CaP nanoparticles that contain layers of calcium phosphate and Toll-like receptor ligand CpG, protecting a core containing the antigenic peptides GagL_85–93_ or gp70_123–141_ (10). We showed that CaP nanoparticles (NP), functionalized with CpG and viral peptides effectively activate DCs *in vitro* and *in vivo* (8–10). To determine, whether DCs produce IFNα after CaP NP uptake, SP DCs were stimulated *in vitro* with CaP NP, functionalized with CpG and viral peptides, and the IFNα response was measured in the supernatant after 4 h. IFNα secretion was strongly induced and detected in the supernatants after stimulation with functionalized CaP NPs compared with untreated DCs (Figure 1A). Importantly, no increase of IFNα levels was detected when CaP NPs were not functionalized with CpG, but instead with the non-immunostimulatory PSS as a substitution for CpG to stabilize the particle structure. These results clearly demonstrate that the IFN I response is dependent on encapsulated CpG and not induced by the NP structure. IFNα was also produced by DCs *in vivo* within the first 3 h after CaP nanoparticle immunization (Figure S2 in Supplementary Material). To investigate whether immunization with CaP NP induces an IFN I response *in vivo*, we analyzed the expression of different of IFN-stimulated genes (ISG) in the skin and the LNs 5 h after subcutaneous immunization. In line with the *in vitro* results, IFNα and several ISGs such as *MX1, IRF7*, and *Usp18* were strongly expressed after immunization with functionalized CaP NPs (Figure 1B). Importantly, expression of ISGs failed after immunization of mice lacking the IFN I receptor (IFNAR^−/−^).

**Figure 2:**
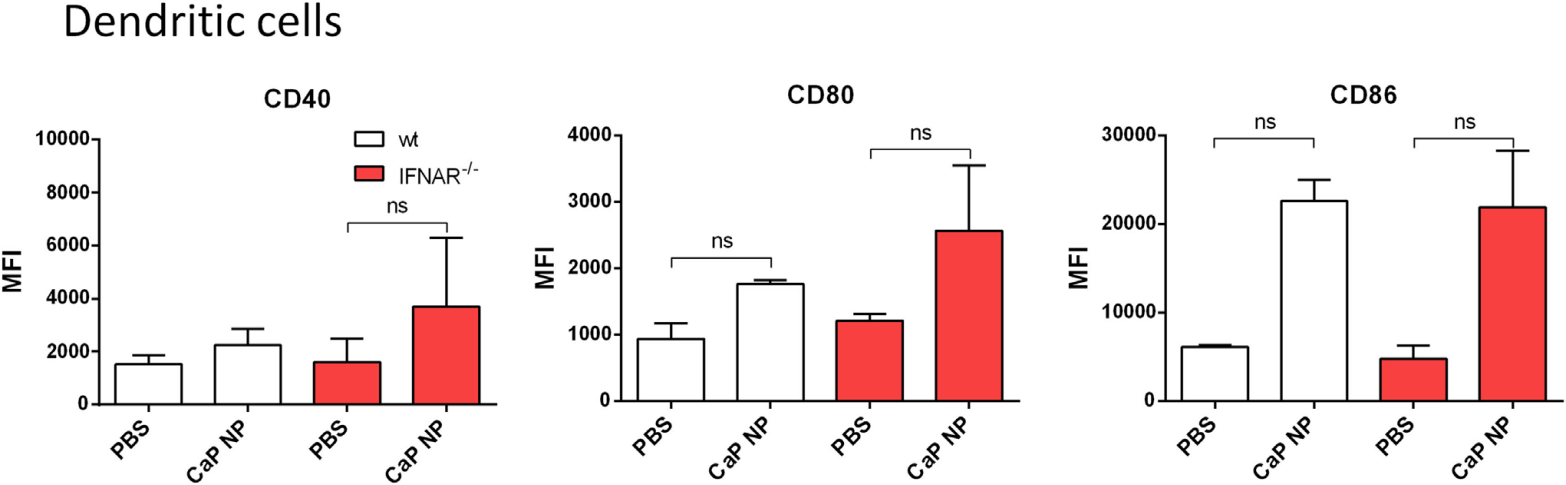
Activation of dendritic cells is independent of type I interferon (IFN I) signaling after vaccination with functionalized calcium phosphate (CaP) nanoparticles (NPs). C57BL/6 mice or IFNAR^−/−^ mice were vaccinated subcutaneously either with phosphate- buffered saline (PBS) or CpG and FV peptide functionalized CaP NPs. 6 h postvaccination, mice were sacrificed and CD11c^high^ cells from the popliteal lymph node were stained for CD40 and expression of costimulatory molecules CD80 and CD86. The results of two independent experiments are depicted, *n* = 6. Data represent mean ± SEM.

We previously showed that the uptake of CaP NP by DCs induced DC maturation (8–10). To test whether the vaccine-induced IFNs I initiated this DC maturation *in vivo*, we immunized wild-type or IFNAR^−/−^ mice with CpG and FV-specific peptide functionalized CaP NPs and analyzed DCs of the draining LN for the expression of costimulatory molecules. Interestingly, both in wild-type as well as in IFNAR^−/−^ mice we observed a strong upregulation of CD40, CD80, and CD86 expression on DCs (Figure 2). These results indicate that the NP mediated maturation of DCs was not dependent on the IFN I response.

### IFNs I Are Important for Initial Priming of Anti-Viral T Cell Responses

**Figure 3:**
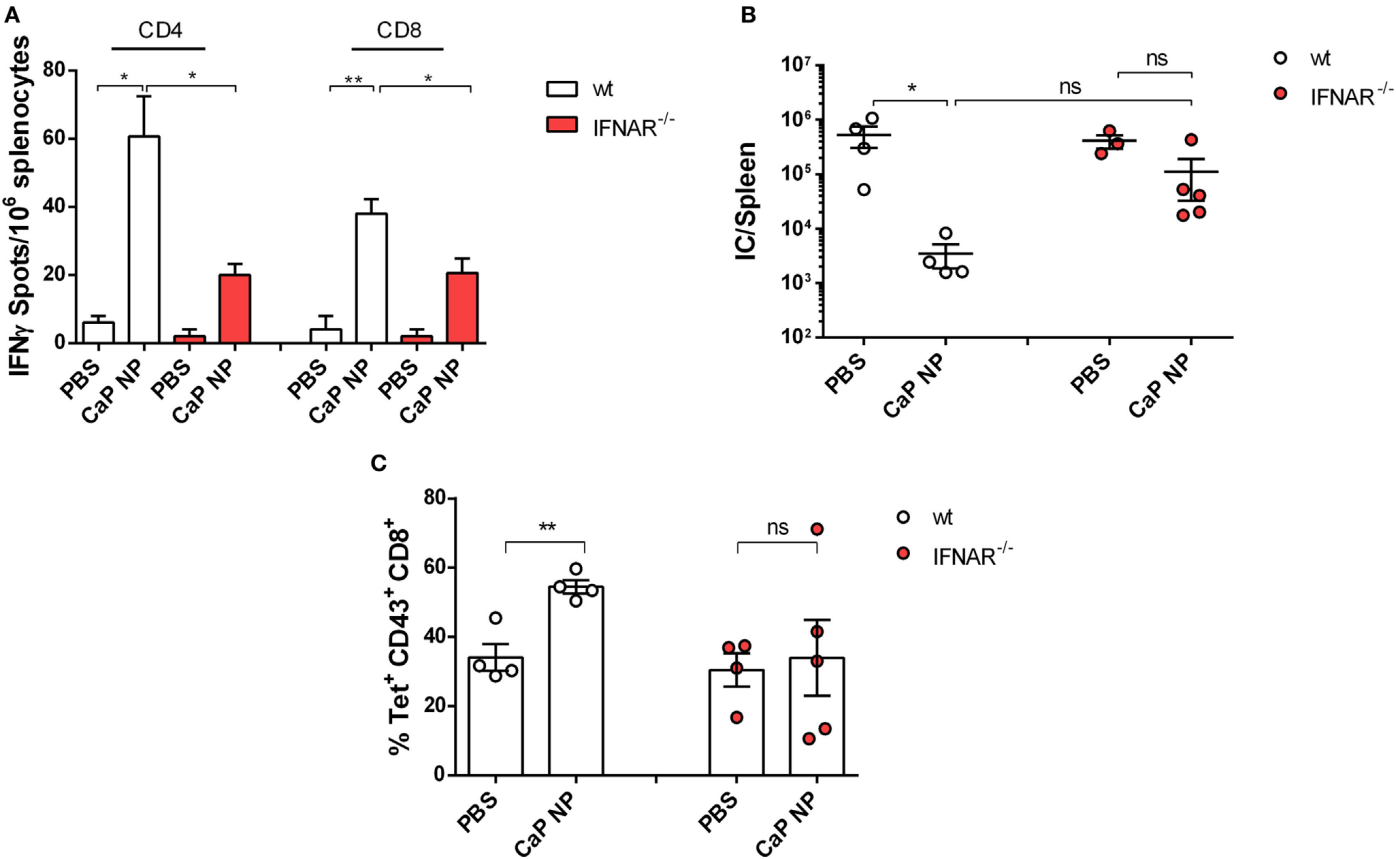
Vaccination induced type I interferon signaling is important for sufficient protection against acute FV infection. **(A)** C57BL/6 mice or IFNAR^−/−^ mice were vaccinated subcutaneously either with phosphate-buffered saline (PBS) or CpG and FV peptide functionalized calcium phosphate (CaP) nanoparticles (NPs) in a prime-boost regiment. 14 days post the last vaccination, mice were sacrificed and splenocytes were restimulated *ex vivo* with 5 µg/mL of GagL_85–93_ or gp70_123–141_ peptide. After 24 h, the numbers of IFN-γ-producing CD4^+^ and CD8^+^ T cells were determined by using an ELISpot reader. The results of two independent experiments are depicted, *n* = 6. Statistical analysis was performed by ANOVA with Bonferroni’s multiple comparisons test (*p < 0.05; **p < 0.01). **(B)** Vaccinated C57BL6 mice or IFNAR^−/−^ mice were challenged with FV 14 days post the last vaccination. 7 days later, mice were sacrificed and infectious centers (ICs) in the spleen were determined. Statistical analysis was performed by Student’s *t*-test (*p < 0.05; **p < 0.01). **(C)** Frequencies of tetramer^+^ FV-specific CD43^+^ CD8^+^ T cells in C57BL6 mice or IFNAR^−/−^ mice 7 days after FV infection. Statistical analysis was performed by Student’s *t*-test (*p < 0.05; **p < 0.01). All data represent mean ± SEM.

To clarify whether induced IFNs I were important for the priming of anti-viral T cell responses, we immunized naïve wild-type or IFNAR^−/−^ mice in a prime-boost regimen with CpG and peptide functionalized CaP NPs and analyzed the numbers of IFNγ producing virus-specific CD4^+^ and CD8^+^ T cells in the spleen. As expected, the numbers of induced FV-specific T cells were strongly elevated in immunized mice compared with PBS treated controls. Importantly, numbers of FV-specific CD4^+^ and CD8^+^ T cells were significantly lower in immunized IFNAR^−/−^ mice in comparison with wild-type mice, indicating that the initial priming of the T cells was impaired in IFNAR^−/−^ mice (Figure 3A). As a consequence, in contrast to immunized wild-type mice, IFNAR^−/−^ mice were not able to control an FV challenge infection (Figure 3B). Whereas immunized wild-type mice showed a significant drop in viral loads, the viral loads in IFNAR^−/−^ mice were unchanged. This result was consistent with reduced numbers of virus-specific MHC-class I H2-D^b^ tetramer^+^ CD8^+^ T cells in prophylactically immunized IFNAR^−/−^ mice in contrast to immunized wild-type mice (Figure 3C).

### Reinforcement of CD8^+^ T Cell Functionality During Chronic Retroviral Infection After NP Immunization Was Dependent on CpG Induced IFNs I

**Figure 4:**
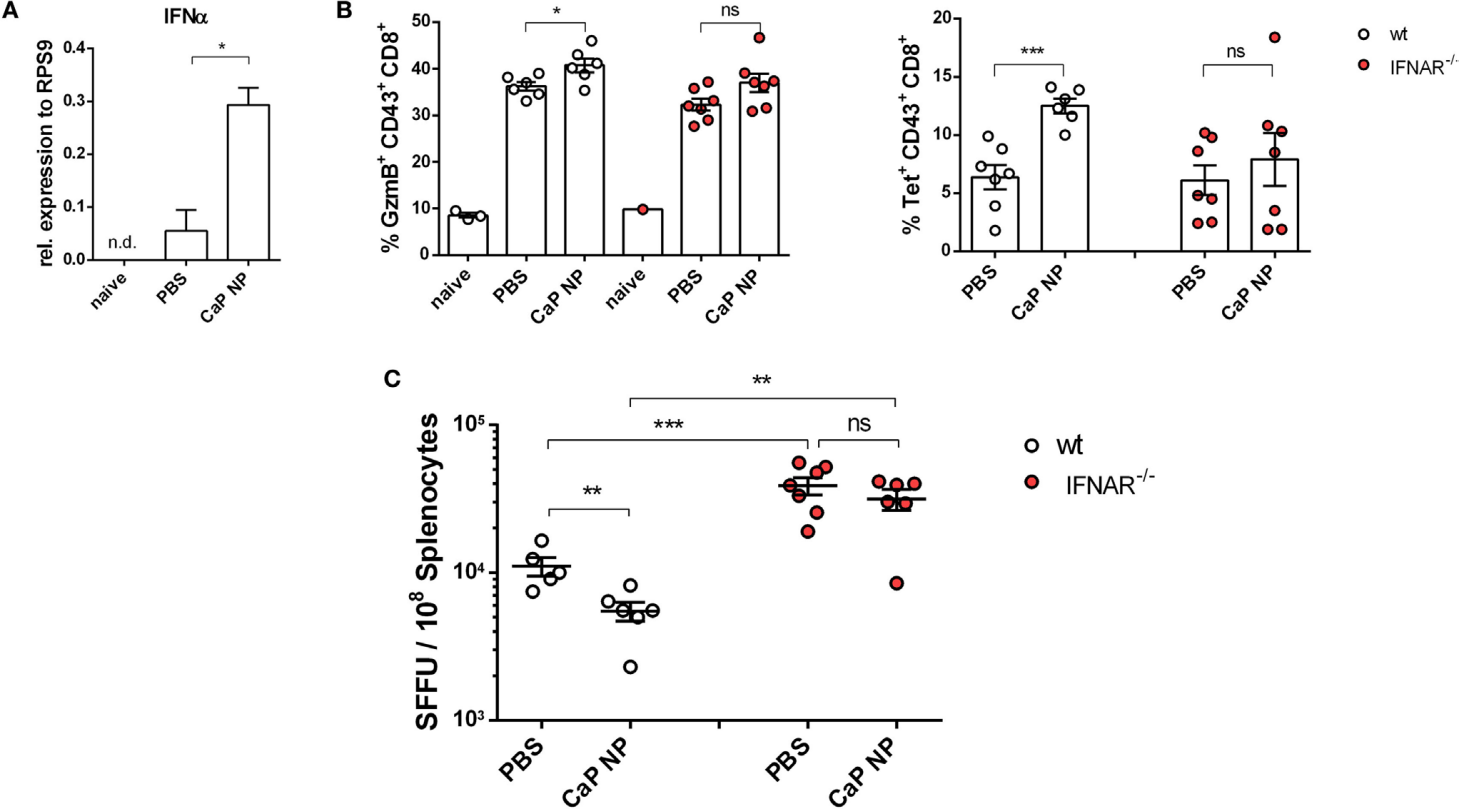
Diminished reactivation of cytolytic CD8^+^ T cells after therapeutic vaccination of chronically infected IFNAR^−/−^ mice. **(A)** Chronically infected C57BL/6 mice were vaccinated subcutaneously either with phosphate-buffered saline (PBS) or CpG and FV peptide functionalized calcium phosphate (CaP) nanoparticles (NPs). 7 days postvaccination RNA was isolated from splenocytes and expression of IFNα was analyzed by quantitative real-time PCR. **(B)** Splenocytes were isolated 7 days postvaccination from non-infected naïve wt or chronically infected wt and IFNAR^−/−^ mice and stained for granzyme B expression in CD43^+^ CD8^+^ T cells and tetramer^+^ FV-specific CD43^+^ CD8^+^ T cells. **(C)** Infectious centers in the spleen were determined 7 days postvaccination. The results of two independent experiments are shown, *n* = 6–7. Bars represent mean ± SEM. Statistical analysis was performed by Student’s *t*-test (*p < 0.05; **p < 0.01; ***p < 0.001).

**Figure 5:**
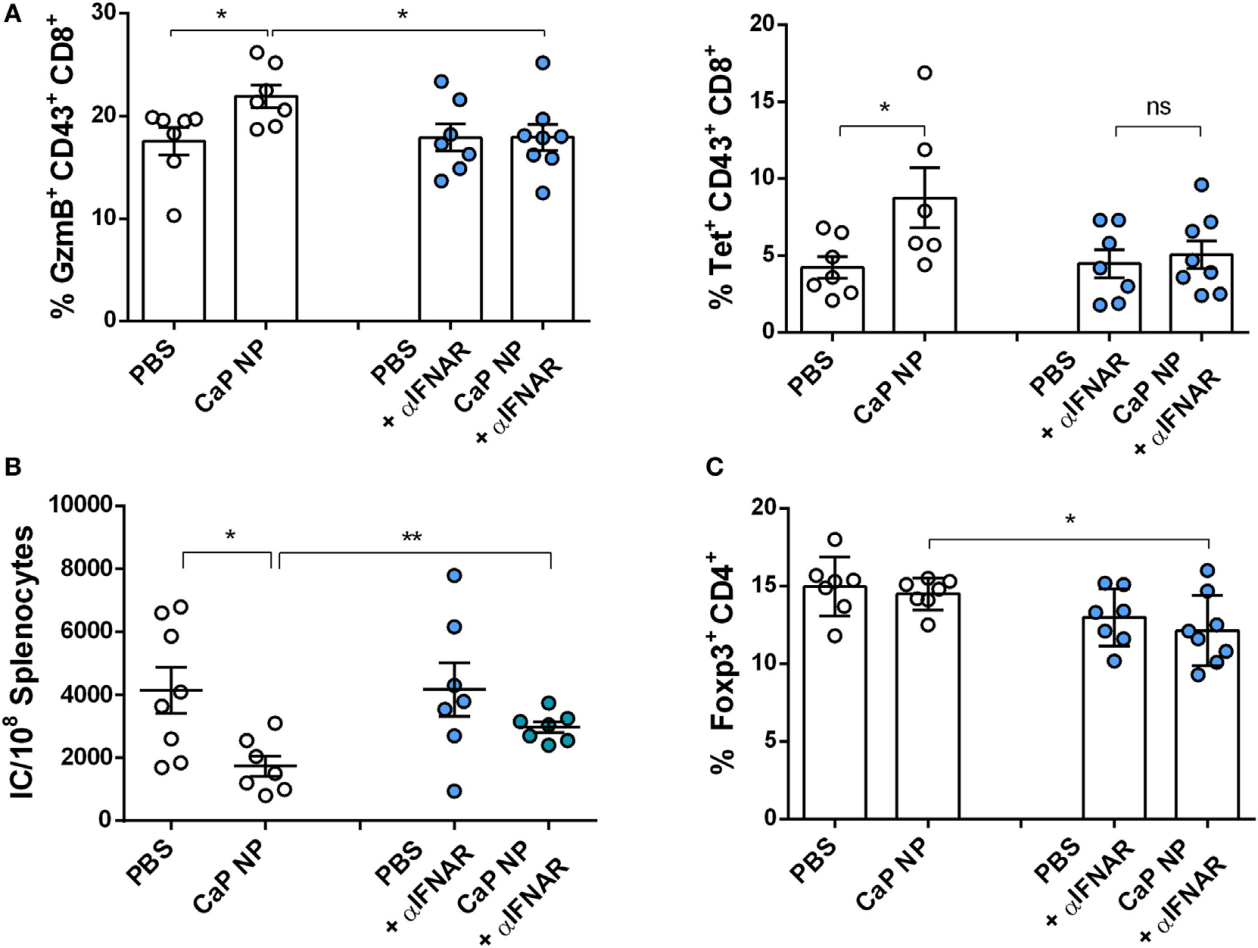
Blocking type I interferon signaling prevents reactivation of cytolytic CD8^+^ T cells. Chronically infected C57BL/6 mice were vaccinated subcutaneously either with phosphate-buffered saline (PBS) or CpG and FV peptide functionalized calcium phosphate (CaP) nanoparticles (NPs). IFNAR blocking monoclonal antibody was applied 1 day before and 2 days after vaccination. **(A)** Splenocytes were isolated 7 days postvaccination and stained for granzyme B expression in CD43^+^ CD8^+^ T cells and tetramer^+^ FV-specific CD43^+^ CD8^+^ T cells. **(B)** Infectious centers in the spleen were determined 7 days postvaccination. **(C)** Splenocytes were stained for Foxp3 expression in CD4^+^ T cells. The results of two independent experiments are shown, *n* = 7–8. Bars represent mean ± SEM. Statistical analysis was performed by Student’s *t*-test (*p < 0.05).

CTLs are crucial for eliminating virus infected cells and controlling the infection. Dysfunctionality of CD8^+^ T cells on the other hand is one important factor in the development of chronic infections. Therefore, the reinforcement of CD8^+^ T cell functionality is the major goal of therapeutic vaccination. Since we demonstrated that IFNs I were essential for efficient CD8^+^ T cell priming, we tested whether IFNs I have also an impact on the reactivation of cytotoxic CD8^+^ T cells and viral clearance in chronically FV infected mice. Chronically infected wild-type and IFNAR^−/−^ mice were therapeutically vaccinated with CpG and peptide functionalized CaP NPs. First, we measured the expression of IFNα in the spleen of vaccinated and unvaccinated chronically FV infected mice. Only low levels of IFNα were detectable in unvaccinated mice, supporting previous findings that FV is a very weak inducer of IFN I responses (38). In contrast, 7 days after vaccination, IFNα expression was significantly elevated compared with unvaccinated mice (Figure 4A). Analyzing the CTL response revealed, that the expression of the cytotoxic molecule GzmB was significantly enhanced in activated CD43^+^ effector CD8^+^ T cells in vaccinated wild-type mice. In contrast, vaccination of IFNAR^−/−^ mice did not increase the frequency of GzmB expressing CD8^+^ T cells (Figure 4B). Furthermore, the unresponsiveness to IFN I signaling inhibited the expansion of activated virus-specific CD8^+^ T cells as their frequency was only elevated in wild-type mice but not in IFNAR^−/−^ mice after therapeutic vaccination (Figure 4B). Well in line with the reduced percentage of virus-specific CD8^+^ T cells in IFNAR^−/−^ mice, immunization of IFNAR^−/−^ mice with functionalized CaP NPs did not reduce the viral loads in contrast to immunization of wild-type mice (Figure 4C). Nevertheless, we noticed that viral loads of chronically infected vaccinated as well as unvaccinated IFNAR^−/−^ mice were significantly higher than in infected wild-type mice. It is known that FV induces only low levels of IFNα early during infection (19). Hence, to proof that the loss in decrease of viral load levels in vaccinated IFNAR^−/−^ mice is not due to an elevated infection level, we just temporarily blocked the IFNα receptor signaling in wild-type mice by injecting a monoclonal antibody before and after vaccination. Importantly, specific blocking of the IFNα receptor signaling combined with vaccination also inhibited the reactivation of cytotoxic GzmB^+^ CD43^+^ CD8^+^ T cells (Figure 5A) and the expansion of activated virus-specific CD8^+^ T cells and impeded the reduction in viral loads after immunization (Figure 5B). Surprisingly, we noticed a decrease in the frequency of Foxp3^+^ Treg when the IFNα receptor was blocked during vaccination (Figure 5C). Taken together, our data clearly demonstrate that the anti-retroviral effect of therapeutic CaP NP vaccination was strongly influenced by the temporary release of IFN I.

### Direct and Indirect Effects of IFNα on CD8^+^ T Cell Functionality

**Figure 6:**
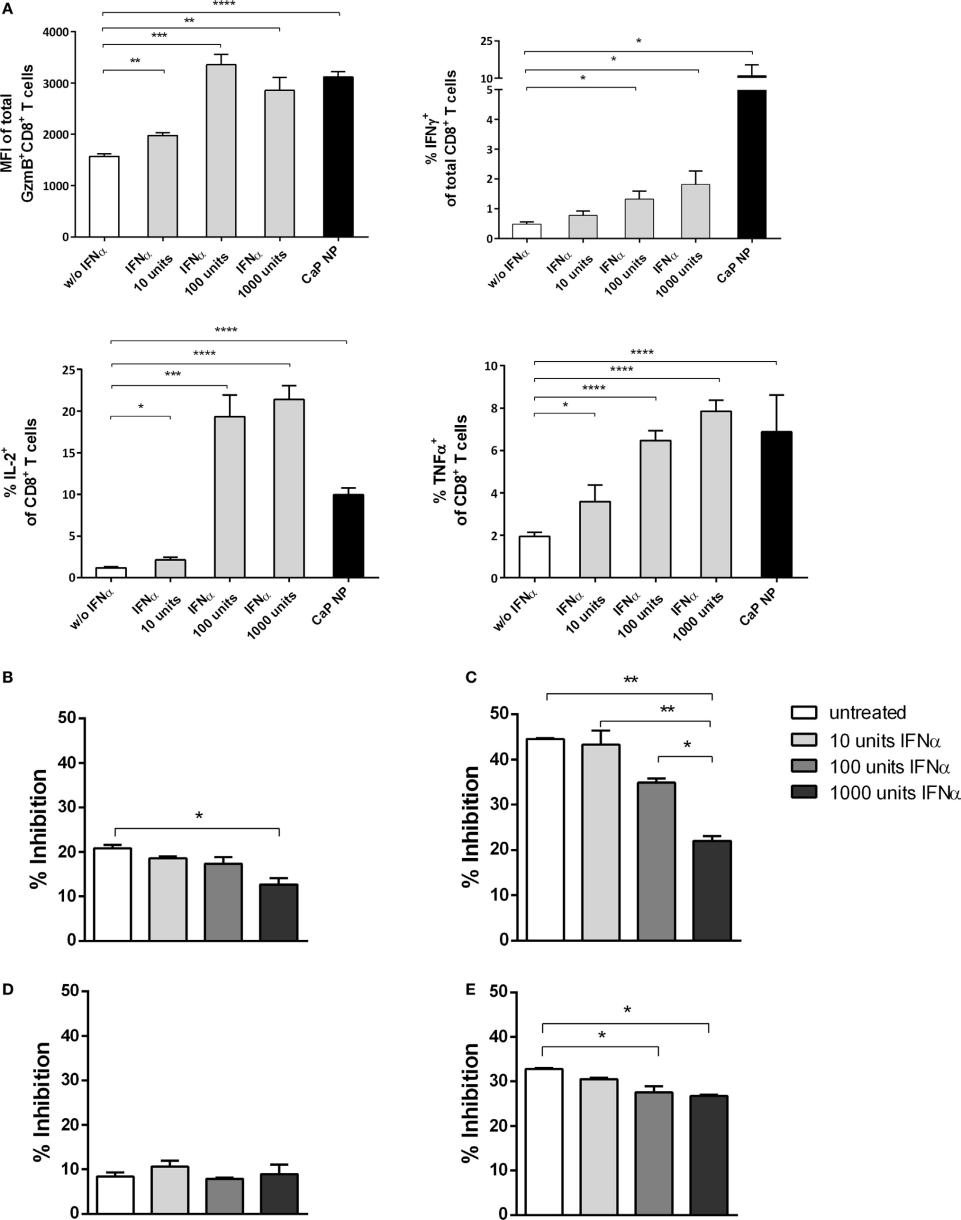
CD8^+^ T cell and regulatory T cells (Treg) functionality is influenced by type I interferon (IFN I). **(A)** GagL peptide pulsed bone-marrow derived dendritic cells were co-cultured with FV-specific CD8^+^ T cells and stimulated with different amounts of universal IFN I. Expression of granzyme B, IFNγ, interleukin-2, and tumor necrosis factor-α (TNFα) was analyzed by intracellular cytokine staining. **(B)** Determination of the influence of IFN I on the suppressive capability of Tregs. Sorted CD4^+^ Foxp3^+^ (eGFP^+^) Treg cells from the spleen of naïve **(B)** or chronically FV infected **(C)** mice were co-cultured with CD8^+^ responder T cells and irradiated antigen-presenting cells (APCs) in the presence of αCD3 and different amounts of universal IFNα. **(D)** CD4^+^ CD25^hi^ Treg cells were sorted from naïve IFNAR^−/−^ mice and co-cultured with wild-type CD8^+^ responder T cells and irradiated IFNAR^−/−^ APCs in the presence of αCD3 and different amounts of universal IFNα. **(E)** CD4^+^ Foxp3^+^ (eGFP^+^) Treg cells from naïve mice were co-cultured with CD8^+^ responder T cells and irradiated APCs both isolated from IFNAR^−/−^ mice in the presence of αCD3 and different amounts of universal IFNα. Proliferation of CD8^+^ T cells was measured by the loss of eFluor dye, and inhibition was calculated. The results of two independent experiments are shown. Bars represent mean ± SEM. Statistical analysis was performed by Student’s *t*-test **(A)** or ANOVA with Bonferroni’s multiple comparisons test **(B–E)** (*p < 0.05; **p < 0.01; ***p < 0.001).

To analyze the underlying mechanisms in more detail, we first tested whether IFNs I acted directly on T cells. Therefore, we stimulated purified CD8^+^ T cells with αCD28 and αCD3 in the presence of different doses of IFNα. As expected, with increasing doses of IFNα, the CD8^+^ T cells showed enhanced effector functions indicated by their capability to express the cytokines IFNγ, IL-2, or TNFα or the cytotoxic molecule GzmB (Figure 6A). Therefore, IFN I seemed to act directly on T cells and enhance their functionality. These results are well in line with recent studies in other viral infection models (17, 39).

To investigate whether IFN I might have an indirect impact on the functionality of CD8^+^ T cells by acting on other cell subsets, we studied the inhibitory capacity of Tregs under the influence of IFNα. We stimulated CD8^+^ T cells alone or in the presence of Foxp3^+^ Tregs, isolated from naïve mice and increasing doses of IFNα (Figure 6; Figure S1 in Supplementary Material). Interestingly, we identified that with increasing concentrations of IFNα the inhibitory capacity of Tregs was significantly decreased (Figure 6B). Furthermore, we repeated the experiment using Tregs from chronically FV infected mice. It was demonstrated previously that Tregs are highly suppressive during chronic FV infection and do suppress CD8^+^ T cell responses (2, 40). Importantly, we observed that even these highly suppressive Tregs were strongly influenced by IFNα, indicated by enhanced CD8^+^ T cell proliferation correlating with increased IFN I doses (Figure 6C). To further confirm that the observed effect is due to the impact of IFNα on Tregs and not on CD8^+^ effector T cells in this experimental setting, we repeated the experiment by either using Tregs or CD8^+^ T cells isolated from IFNAR^−/−^ mice. Well in line with the former results, the inhibitory capacity was unaffected when IFNAR^−/−^ Tregs were used and still significantly decreased when CD8^+^ T cells were unresponsive to IFN I (Figures 6D,E). Therefore, these results imply a direct and indirect effect of vaccination induced IFN I on the CD8^+^ T cell response with a close connection between enhanced functionality of cytotoxic CD8^+^ T cells and the altered suppressor capacity of Tregs.

## Discussion

Novel strategies for therapeutic treatment of chronic viral infections are still of great interest since a protective vaccine against, e.g., HIV is still elusive. CaP NPs are an established tool for the transportation of small molecules across cell membranes and the sufficient induction of anti-viral immune responses, as they can get functionalized with several kinds of small molecules (7, 8). We recently proved their potential as therapeutic anti-retroviral vaccine. Immunization with CaP NPs functionalized with FV-derived T cell epitopes and the TLR9 ligand CpG leads to a significant reactivation of CD4^+^ and CD8^+^ T cell responses, which improves the eradication of infected cells during chronic FV infection (10, 11). Therefore, CaP NPs are efficient carriers for immunotherapeutic molecules. However, so far, it was unclear which of the specific vaccination induced factors are predominantly responsible for the observed positive effects. In the current study, we determined the impact of immunotherapeutic CaP NP vaccination on the reactivation of the immune response. Since CpG ODN is an inducer of IFN I, we studied the effect of vaccine mediated IFN I release on the CD8^+^ T cell immunity. Experiments in both, IFNAR^−/−^ mice and wild-type mice treated with an IFNAR blocking antibody during therapeutic NP vaccination, revealed an essential role for IFN I in reinforcing the CTL functionality and controlling chronic FV infection. This finding is very interesting, since the role of IFN I during viral infection is discussed controversially. While IFN I where shown to enhance T cell priming and increase the cytotoxic capacity of CD8^+^ T cells during viral infections (14–17), they can also foster mechanisms of immunosuppression (25, 41). In the current study, we demonstrated that IFNα strongly enhances the functionality of CD8^+^ T cells *in vitro*. Increasing amounts of IFNα led to the enhanced production of pro-inflammatory cytokines such as IL-2 and IFNy. Interestingly, stimulation of DC/T cell co-cultures with CpG functionalized CaP NPs led to the activation of CD8^+^ T cells comparable with stimulation of cells in the presence of 100 U of IFNα. This probably gives an idea about IFN I levels after CaP NP vaccination *in vivo*. Previously, it was demonstrated that the induction of IFN I can be essential for the efficacy of vaccination (42). However, recent reports proposed also negative effects of constant IFN I signaling especially in the context of chronic viral infections, since it mediates immune dysfunction during persistent LCMV or chronic HIV infection of humanized mice (24, 25, 43). Prior studies in the LCMV model reported no effect of exogenous IFN I on the immune response at later time points of persistent infection (41), while the treatment of HIV-infected patients with exogenous IFNα can control viral onset (44–47). Interestingly, in the FV model vaccination induced IFN I turned out to be crucial for the successful reactivation of CD8^+^ T cells and effective virus clearance. After CaP NP vaccination of IFNAR^−/−^ mice or even after blockade of IFNAR we did not see an elevated reactivation of the cytolytic CD8^+^ T cell response nor did the interference of the IFN I signaling lead to an enhanced elimination of virus infected cells as proposed in persistently LCMV infected mice (25). In contrast, unresponsiveness to IFN I consequently resulted in an insufficient virus clearance after therapeutic NP vaccination. Timing and magnitude of the IFN I response can have severe effects on CD8^+^ T cell exhaustion (41), and the diverse IFN I signature in FV, LCMV, and HIV infection could influence the immune response differently. Primary FV induced IFN I levels are weak during the early phase of infection (19). For example, IFNα levels are approximately 10 times lower during the first 24 h compared with the reported levels in acute LCMV infection. Thus, the immune response during FV infection might not be as exposed to IFN I as during LCMV infection, which could alter the responsiveness of immune cells to IFN I during the chronic phase of infection. This finding demonstrates that the impact of IFN I on the anti-viral immune response strongly depends on the virus infection.

Furthermore Tregs are known to efficiently downregulate the virus-specific CD8^+^ T cell response during chronic FV infection (2, 40). However, the detailed suppressive mechanisms are still not fully understood. We now show that the suppressive capacity of Tregs during chronic FV infection can be influenced by IFN I. The inhibitory capacity of Tregs was decreased with increasing IFNα doses indicated by elevated CD8^+^ T cell proliferation. Experiments using either CD8^+^ T cells or Tregs from IFNAR^−/−^ mice confirmed that IFNα acts exclusively on Tregs in this experimental setup. That IFN I regulates the functionality of Tregs was reported previously (48). However, the effect of IFNα on highly active Tregs from chronically infected mice has not been tested so far. Therefore, our results suggest that vaccination induced IFN I is not only important for the direct but also indirect reactivation of CD8^+^ T cells by influencing the functionality of Tregs.

Taken together, our results demonstrate a significant impact of IFN I for the efficacy of therapeutic vaccination during chronic FV infection. We propose a direct and indirect effect of vaccination induced IFN I on the functionality of CD8^+^ T cells by influencing the inhibitory capacity of Tregs. Our data suggest a key role for IFN I in modulating the immune response during therapeutic vaccination in chronic viral infection.

## Ethics Statement

This study was carried out in accordance with the recommendations of the Society for Laboratory Animal Science (GV-SOLAS) and the European Health Law of the Federation of Laboratory Animal Science Associations (FELASA). The protocol was approved by the North Rhine-Westphalia State Agency for Nature, Environment and Consumer Protection (LANUF), Germany (Permit Number: Az.: 84-02.04.2014.A290).

## Author Contributions

AW, JB, ME, and TK conceived and designed the experiments. TK, JD, and SK performed the experiments. TK, AW, UD, WB, WH, and ME analyzed the data and reviewed and edited the manuscript. OR, SK, ME, WB, KS, and KL contributed reagents/materials/analysis tools. TK and AW wrote the paper.

## Conflict of Interest Statement

The authors declare that the research was conducted in the absence of any commercial or financial relationships that could be construed as a potential conflict of interest.

## References

[1] Barber DL Wherry EJ Masopust D Zhu B Allison JP Sharpe AH Restoring function in exhausted CD8 T cells during chronic viral infection. Nature (2006) 439:682–7. 10.1038/nature04444 16382236

[2] Dietze KK Zelinskyy G Gibbert K Schimmer S Francois S Myers L Transient depletion of regulatory T cells in transgenic mice reactivates virus-specific CD8+ T cells and reduces chronic retroviral set points. Proc Natl Acad Sci U S A (2011) 108:2420–5. 10.1073/pnas.1015148108 21262821 PMC3038736

[3] Trautmann L Kill: boosting HIV-specific immune responses. Curr Opin HIV AIDS (2016) 11:409–16. 10.1097/COH.0000000000000286 27054280 PMC5098478

[4] Sultan H Fesenkova VI Addis D Fan AE Kumai T Wu J Designing therapeutic cancer vaccines by mimicking viral infections. Cancer Immunol Immunother (2017) 66:203–13. 10.1007/s00262-016-1834-5 27052572 PMC5053837

[5] Barouch DH Deeks SG Immunologic strategies for HIV-1 remission and eradication. Science (2014) 345:169–74. 10.1126/science.1255512 25013067 PMC4096716

[6] Cillo AR Mellors JW Which therapeutic strategy will achieve a cure for HIV-1?. Curr Opin Virol (2016) 18:14–9. 10.1016/j.coviro.2016.02.001 26985878

[7] Sokolova V Rotan O Klesing J Nalbant P Buer J Knuschke T Calcium phosphate nanoparticles as versatile carrier for small and large molecules across cell membranes. J Nanopart Res (2012) 14:910 10.1007/s11051-012-0910-9

[8] Sokolova V Knuschke T Kovtun A Buer J Epple M Westendorf AM The use of calcium phosphate nanoparticles encapsulating toll-like receptor ligands and the antigen hemagglutinin to induce dendritic cell maturation and T cell activation. Biomaterials (2010) 31:5627–33. 10.1016/j.biomaterials.2010.03.067 20417963

[9] Knuschke T Sokolova V Rotan O Wadwa M Tenbusch M Hansen W Immunization with biodegradable nanoparticles efficiently induces cellular immunity and protects against influenza virus infection. J Immunol (2013) 190:6221–9. 10.4049/jimmunol.1202654 23667109

[10] Knuschke T Bayer W Rotan O Sokolova V Wadwa M Kirschning CJ Prophylactic and therapeutic vaccination with a nanoparticle-based peptide vaccine induces efficient protective immunity during acute and chronic retroviral infection. Nanomedicine (2014) 10:1787–98. 10.1016/j.nano.2014.06.014 25014891

[11] Knuschke T Rotan O Bayer W Sokolova V Hansen W Sparwasser T Combination of nanoparticle-based therapeutic vaccination and transient ablation of regulatory T cells enhances anti-viral immunity during chronic retroviral infection. Retrovirology (2016) 13:24 10.1186/s12977-016-0258-9 27076190 PMC4831142

[12] Takeda K Akira S Toll-like receptors in innate immunity. Int Immunol (2005) 17:1–14. 10.1093/intimm/dxh186 15585605

[13] Welsh RM Bahl K Marshall HD Urban SL Type 1 interferons and antiviral CD8 T-cell responses. PLoS Pathog (2012) 8:e1002352 10.1371/journal.ppat.1002352 22241987 PMC3252364

[14] Muller U Steinhoff U Reis LF Hemmi S Pavlovic J Zinkernagel RM Functional role of type I and type II interferons in antiviral defense. Science (1994) 264:1918–21. 10.1126/science.8009221 8009221

[15] Montoya M Schiavoni G Mattei F Gresser I Belardelli F Borrow P Type I interferons produced by dendritic cells promote their phenotypic and functional activation. Blood (2002) 99:3263–71. 10.1182/blood.V99.9.3263 11964292

[16] Curtsinger JM Valenzuela JO Agarwal P Lins D Mescher MF Type I IFNs provide a third signal to CD8 T cells to stimulate clonal expansion and differentiation. J Immunol (2005) 174:4465–9. 10.4049/jimmunol.174.8.4465 15814665

[17] Kolumam GA Thomas S Thompson LJ Sprent J Murali-Krishna K Type I interferons act directly on CD8 T cells to allow clonal expansion and memory formation in response to viral infection. J Exp Med (2005) 202:637–50. 10.1084/jem.20050821 16129706 PMC2212878

[18] Feldman S Stein D Amrute S Denny T Garcia Z Kloser P Decreased interferon-alpha production in HIV-infected patients correlates with numerical and functional deficiencies in circulating type 2 dendritic cell precursors. Clin Immunol (2001) 101:201–10. 10.1006/clim.2001.5111 11683579

[19] Gerlach N Schimmer S Weiss S Kalinke U Dittmer U Effects of type I interferons on Friend retrovirus infection. J Virol (2006) 80:3438–44. 10.1128/JVI.80.7.3438-3444.2006 16537611 PMC1440373

[20] Cha L Berry CM Nolan D Castley A Fernandez S French MA Interferon-alpha, immune activation and immune dysfunction in treated HIV infection. Clin Transl Immunology (2014) 3:e10 10.1038/cti.2014.1 25505958 PMC4232062

[21] Lavender KJ Gibbert K Peterson KE Van Dis E Francois S Woods T Interferon alpha subtype-specific suppression of HIV-1 infection in vivo. J Virol (2016) 90:6001–13. 10.1128/JVI.00451-16 27099312 PMC4907223

[22] Harper MS Guo K Gibbert K Lee EJ Dillon SM Barrett BS Interferon-alpha subtypes in an ex vivo model of acute HIV-1 infection: expression, potency and effector mechanisms. PLoS Pathog (2015) 11:e1005254 10.1371/journal.ppat.1005254 26529416 PMC4631339

[23] Sandler NG Bosinger SE Estes JD Zhu RT Tharp GK Boritz E Type I interferon responses in rhesus macaques prevent SIV infection and slow disease progression. Nature (2014) 511:601–5. 10.1038/nature13554 25043006 PMC4418221

[24] Teijaro JR Ng C Lee AM Sullivan BM Sheehan KC Welch M Persistent LCMV infection is controlled by blockade of type I interferon signaling. Science (2013) 340:207–11. 10.1126/science.1235214 23580529 PMC3640797

[25] Wilson EB Yamada DH Elsaesser H Herskovitz J Deng J Cheng G Blockade of chronic type I interferon signaling to control persistent LCMV infection. Science (2013) 340:202–7. 10.1126/science.1235208 23580528 PMC3704950

[26] Zelinskyy G Robertson SJ Schimmer S Messer RJ Hasenkrug KJ Dittmer U CD8+ T-cell dysfunction due to cytolytic granule deficiency in persistent Friend retrovirus infection. J Virol (2005) 79:10619–26. 10.1128/JVI.79.16.10619-10626.2005 16051854 PMC1182617

[27] Gibbert K Dietze KK Zelinskyy G Lang KS Barchet W Kirschning CJ Polyinosinic-polycytidylic acid treatment of Friend retrovirus-infected mice improves functional properties of virus-specific T cells and prevents virus-induced disease. J Immunol (2010) 185:6179–89. 10.4049/jimmunol.1000858 20943997

[28] Hwang SY Hertzog PJ Holland KA Sumarsono SH Tymms MJ Hamilton G A null mutation in the gene encoding a type I interferon receptor component eliminates antiproliferative and antiviral responses to interferons alpha and beta and alters macrophage responses. Proc Natl Acad Sci U S A (1995) 92:11284–8. 7479980 10.1073/pnas.92.24.11284 PMC40616

[29] Lahl K Loddenkemper C Drouin C Freyer J Arnason J Eberl G Selective depletion of Foxp3+ regulatory T cells induces a scurfy-like disease. J Exp Med (2007) 204:57–63. 10.1084/jem.20061852 17200412 PMC2118432

[30] Lander MR Chattopadhyay SK A Mus dunni cell line that lacks sequences closely related to endogenous murine leukemia viruses and can be infected by ectropic, amphotropic, xenotropic, and mink cell focus-forming viruses. J Virol (1984) 52:695–8. 6092693 10.1128/jvi.52.2.695-698.1984 PMC254577

[31] Sokolova V Knuschke T Buer J Westendorf AM Epple M Quantitative determination of the composition of multi-shell calcium phosphate-oligonucleotide nanoparticles and their application for the activation of dendritic cells. Acta Biomater (2011) 7:4029–36. 10.1016/j.actbio.2011.07.010 21784177

[32] Chen W Qin H Chesebro B Cheever MA Identification of a gag-encoded cytotoxic T-lymphocyte epitope from FBL-3 leukemia shared by Friend, Moloney, and Rauscher murine leukemia virus-induced tumors. J Virol (1996) 70:7773–82. 8892898 10.1128/jvi.70.11.7773-7782.1996 PMC190847

[33] Balkow S Krux F Loser K Becker JU Grabbe S Dittmer U Friend retrovirus infection of myeloid dendritic cells impairs maturation, prolongs contact to naive T cells, and favors expansion of regulatory T cells. Blood (2007) 110:3949–58. 10.1182/blood-2007-05-092189 17699743

[34] Li JP DAndrea AD Lodish HF Baltimore D Activation of cell growth by binding of Friend spleen focus-forming virus gp55 glycoprotein to the erythropoietin receptor. Nature (1990) 343:762–4. 10.1038/343762a0 2154701

[35] Chesebro B Wehrly K Stimpfling J Host genetic control of recovery from Friend leukemia virus-induced splenomegaly: mapping of a gene within the major histocompatability complex. J Exp Med (1974) 140:1457–67. 10.1084/jem.140.6.1457 4430891 PMC2139745

[36] Dittmer U Brooks DM Hasenkrug KJ Characterization of a live-attenuated retroviral vaccine demonstrates protection via immune mechanisms. J Virol (1998) 72:6554–8. 9658099 10.1128/jvi.72.8.6554-6558.1998 PMC109828

[37] Robertson MN Miyazawa M Mori S Caughey B Evans LH Hayes SF Production of monoclonal antibodies reactive with a denatured form of the Friend murine leukemia virus gp70 envelope protein: use in a focal infectivity assay, immunohistochemical studies, electron microscopy and western blotting. J Virol Methods (1991) 34:255–71. 10.1016/0166-0934(91)90105-9 1744218

[38] Gibbert K Joedicke JJ Meryk A Trilling M Francois S Duppach J Interferon-alpha subtype 11 activates NK cells and enables control of retroviral infection. PLoS Pathog (2012) 8:e1002868 10.1371/journal.ppat.1002868 22912583 PMC3415439

[39] Jennings RN Grayson JM Barton ES Type I interferon signaling enhances CD8+ T cell effector function and differentiation during murine gammaherpesvirus 68 infection. J Virol (2014) 88:14040–9. 10.1128/JVI.02360-14 25253356 PMC4249115

[40] Dietze KK Zelinskyy G Liu J Kretzmer F Schimmer S Dittmer U Combining regulatory T cell depletion and inhibitory receptor blockade improves reactivation of exhausted virus-specific CD8+ T cells and efficiently reduces chronic retroviral loads. PLoS Pathog (2013) 9:e1003798 10.1371/journal.ppat.1003798 24339778 PMC3855586

[41] Wang Y Swiecki M Cella M Alber G Schreiber RD Gilfillan S Timing and magnitude of type I interferon responses by distinct sensors impact CD8 T cell exhaustion and chronic viral infection. Cell Host Microbe (2012) 11:631–42. 10.1016/j.chom.2012.05.003 22704623 PMC3572910

[42] Kranz LM Diken M Haas H Kreiter S Loquai C Reuter KC Systemic RNA delivery to dendritic cells exploits antiviral defence for cancer immunotherapy. Nature (2016) 534:396–401. 10.1038/nature18300 27281205

[43] Zhen A Rezek V Youn C Lam B Chang N Rick J Targeting type I interferon-mediated activation restores immune function in chronic HIV infection. J Clin Invest (2017) 127:260–8. 10.1172/JCI89488 27941243 PMC5199686

[44] Asmuth DM Murphy RL Rosenkranz SL Lertora JJ Kottilil S Cramer Y Safety, tolerability, and mechanisms of antiretroviral activity of pegylated interferon Alfa-2a in HIV-1-monoinfected participants: a phase II clinical trial. J Infect Dis (2010) 201:1686–96. 10.1086/652420 20420510 PMC2946345

[45] Azzoni L Foulkes AS Papasavvas E Mexas AM Lynn KM Mounzer K Pegylated Interferon alfa-2a monotherapy results in suppression of HIV type 1 replication and decreased cell-associated HIV DNA integration. J Infect Dis (2013) 207:213–22. 10.1093/infdis/jis663 23105144 PMC3532820

[46] Hubbard JJ Greenwell-Wild T Barrett L Yang J Lempicki RA Wahl SM Host gene expression changes correlating with anti-HIV-1 effects in human subjects after treatment with peginterferon Alfa-2a. J Infect Dis (2012) 205:1443–7. 10.1093/infdis/jis211 22454462 PMC3324397

[47] Pillai SK Abdel-Mohsen M Guatelli J Skasko M Monto A Fujimoto K Role of retroviral restriction factors in the interferon-alpha-mediated suppression of HIV-1 in vivo. Proc Natl Acad Sci U S A (2012) 109:3035–40. 10.1073/pnas.1111573109 22315404 PMC3286922

[48] Pace L Vitale S Dettori B Palombi C La Sorsa V Belardelli F APC activation by IFN-alpha decreases regulatory T cell and enhances Th cell functions. J Immunol (2010) 184:5969–79. 10.4049/jimmunol.0900526 20427775

