# Supplementary material for: Induction of Type I Interferons by Therapeutic Nanoparticle-Based Vaccination Is Indispensable to Reinforce Cytotoxic CD8+ T Cell Responses During Chronic Retroviral Infection
Source: Front Immunol. 2018 Apr 23;9:614. doi: 10.3389/fimmu.2018.00614 (PMC5924795; doi:10.3389/fimmu.2018.00614)
Supplement: Supplementary file 1 [file data_sheet_1.PDF]

Supplementary Material

Induction of type I interferons by therapeutic nanoparticle-based vaccination is indispensable to reinforce cytotoxic $CD8^+$ T cell responses during chronic retroviral infection

Torben Knuschke[1]*, Olga Rotan[2], Wibke Bayer[3], Sebastian Kollenda[2], Julia Dickow[3], Kathrin Sutter[3], Wiebke Hansen[1], Ulf Dittmer[3], Karl S. Lang[4], Matthias Epple[2], Jan Buer[1], Astrid M. Westendorf[1]

*** Correspondence:** Torben Knuschke: torben.knuschke@uk-essen.de

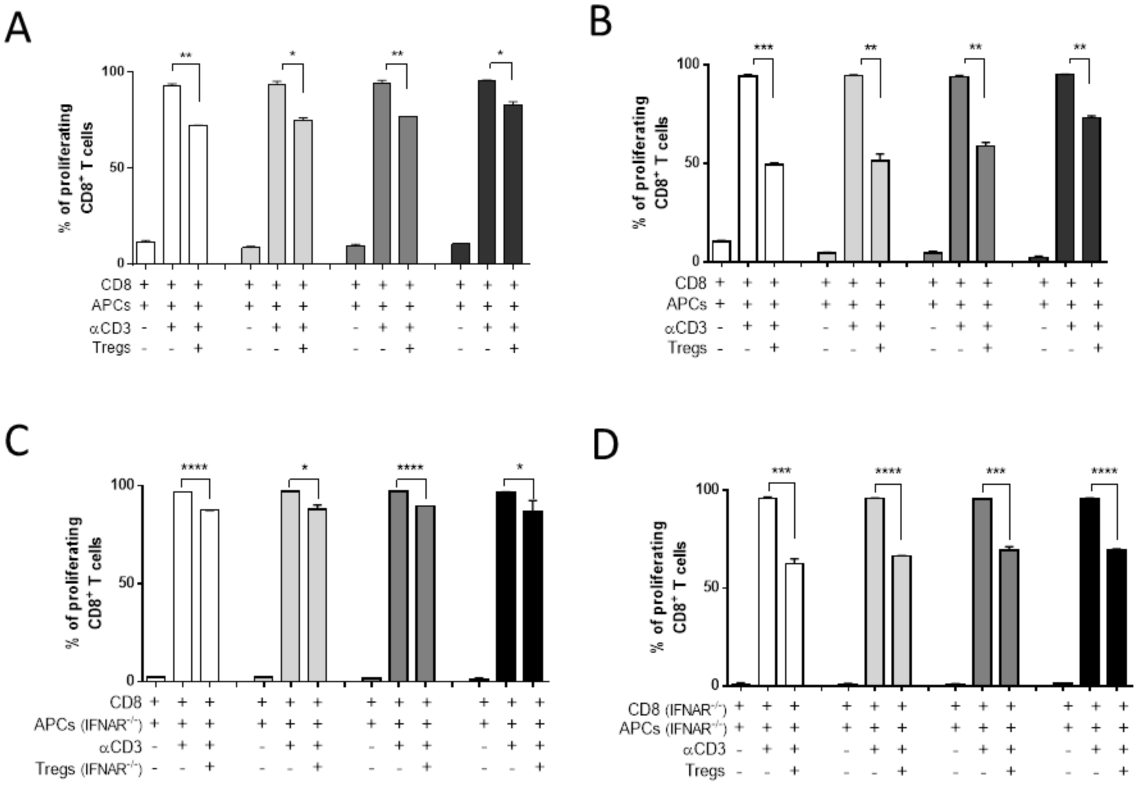

Supplementary Figure 1. Determination of the influence of IFN I on the suppressive capability of Tregs. Sorted CD4$^+$ Foxp3$^+$ (eGFP$^+$) Treg cells from the spleen of naïve (A) or chronically FV infected (B) mice or CD4$^+$ CD25^{hi} Treg cells from naïve IFNAR$^{-/-}$ mice (C) were co-cultured with CD8$^+$ responder T cells and irradiated antigen presenting cells (APCs) in the presence or absence of αCD3 and different amounts of universal IFNα. D) CD4$^+$ Foxp3$^+$ (eGFP$^+$) Treg cells from naïve mice were co-cultured with CD8$^+$ responder T cells and irradiated APCs both isolated from IFNAR$^{-/-}$ mice in the presence or absence of αCD3 and different amounts of universal IFNα. Proliferation of CD8$^+$ T cells was measured by the loss of eFluor dye. The results of 2 independent experiments are shown. Bars represent mean ± SEM. Statistical analysis was performed by ANOVA with Bonferroni's multiple comparisons test (*$p<0.05$; ***$p<0.001$; ****$p<0.0001$).

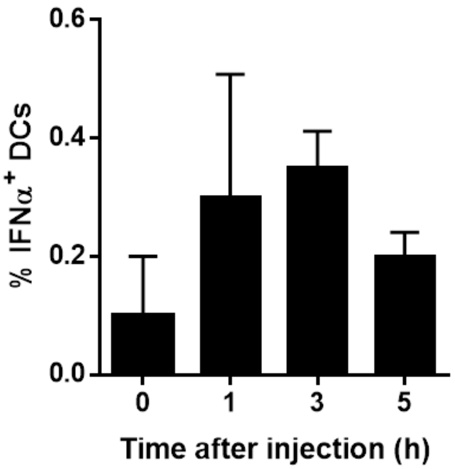

Supplementary Figure 2: IFNα is secreted by DCs *in vivo* after CaP nanoparticle vaccination. C57BL/6 mice were vaccinated subcutaneously with CpG and FV peptide functionalized CaP NPs. Afterwards, mice were sacrificed and $CD11c^{+}$ $F4/80^{-}$ cells from the popliteal lymph node were stained for IFNα after incubation in the presence of brefeldin A. The results of 2 independent experiments are depicted. n = 4. Data represent mean ± SEM.
